# Exploring Quinazoline as a Scaffold for Developing Novel Therapeutics in Alzheimer’s Disease

**DOI:** 10.3390/molecules30030555

**Published:** 2025-01-26

**Authors:** Qais Abualassal, Zead Abudayeh, Ala’ Sirhan, Abdulrahman Mkia

**Affiliations:** 1 Department of Applied Pharmaceutical Sciences and Clinical Pharmacy, Faculty of Pharmacy, Isra University, Queen Alia International Airport Street, Amman 11622, Jordan; zead.abudayeh@iu.edu.jo; 2 Department of Pharmacy, Faculty of Pharmacy, Amman Arab University, Amman 11953, Jordan; a.sirhan@aau.edu.jo; 3 Department of Biotechnology, Faculty of Allied Medical Sciences, Al-Ahliyya Amman University, Amman 19328, Jordan; a.mkia@ammanu.edu.jo

**Keywords:** quinazoline, Alzheimer’s disease, drug discovery, cholinergic hypothesis, beta-amyloid hypothesis

## Abstract

Quinazoline, a privileged scaffold in medicinal chemistry, offers promising potential in the synthesis of anti-Alzheimer’s disease (AD) drugs. This heterocyclic compound, characterized by its fused benzene and pyrimidine rings, enables the design of multifunctional agents targeting AD pathology. The drug-like aspects and pharmaceutical features of quinazoline derivatives have the potential to give rise to various therapeutic drugs. AD is a progressive neurodegenerative condition marked by memory decline, cognitive deterioration, and language disorders. Given its complexity and multifaceted nature, there is a pressing need to discover multi-target drugs to effectively address this debilitating disorder. A comprehensive literature review has demonstrated that quinazoline derivatives exhibit a wide range of therapeutic potential for AD. These compounds function as inhibitors of cholinesterases, β-amyloid aggregation, oxidative stress, and tau protein, among other protective effects. Here, we highlight the most significant and recent research on quinazoline-based anti-AD agents, aiming to support the development and discovery of novel treatments for AD.

## 1. Introduction 

**Figure 1:**
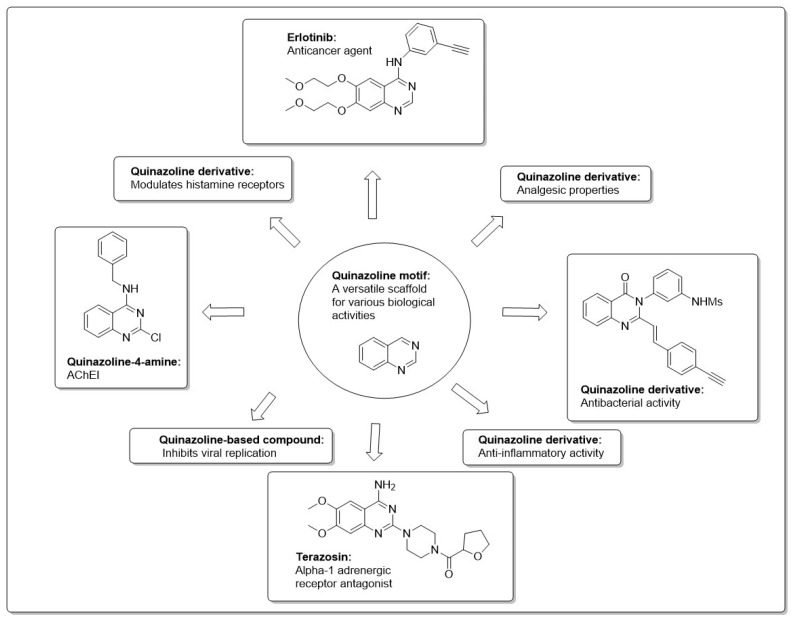
Some quinazoline derivatives and their medicinal activity.

The development of quinazoline derivatives was initiated with the identification of febrifugine, a quinazolinone alkaloid extracted from the Chinese medicinal plant Dichroa febrifuga, known for its reported antimalarial properties, which spurred interest in the development and study of quinazoline derivatives for various therapeutic applications [1]. Quinazoline (1,3-diazanaphthalene) is a yellow crystalline compound first synthesized by George Gabriel in 1903. However, the initial derivative of quinazoline was prepared by Griess 34 years before that [2]. The quinazoline moiety is a heterocyclic compound comprising two fused six-member rings: a benzene ring and a pyrimidine ring. The presence of the fused benzene ring significantly alters the properties of the pyrimidine ring (Figure 1). The properties of substituted quinazolines depend largely on the nature of the substituents and their position in the pyrimidine ring or benzene ring. Substituents can influence electronic, steric, and biological activity [3,4].

Currently, over 200 naturally occurring quinazoline alkaloids have been extracted from a variety of sources, including numerous plant families, as well as animals and microorganisms. These naturally occurring compounds have inspired the development of numerous synthetic derivatives, which have been explored for various medicinal applications [5]. Research and development on the biological activity of quinazoline agents gained significant attention with the development of derivatives like 2-methyl-1, 3-aryl-4-quinazolines [6]. Over the last decades, numerous clinical drugs with the quinazoline structure as their principal nucleus have been synthesized and studied for their biological activities [7,8]. These studies helped establish quinazolines as a valuable scaffold in medicinal chemistry, leading to the development of various drugs with diverse therapeutic applications, like neurodegenerative [9], anticancer [10], anti-microbial [11], antiviral [12], antiallergic [13], anti-hypertensive [14] analgesic [15], and anti-inflammatory applications [16], among others. Figure 1 showcases several notable pharmaceutical compounds featuring quinazoline structures and their activities.

The diverse biological activities of the quinazoline nucleus have captivated the interest of medicinal chemists, prompting extensive exploration of this scaffold for its potential in treating various diseases. A vast number of synthetic routes have been developed [17].For instance, prazosin and terazosin are prescribed for treating benign prostatic hyperplasia (BPH), and prazosin is additionally used to manage post-traumatic stress disorder (PTSD) [18]. Other quinazoline derivatives have been used as active components in medications, such as proquazone, which is a non-steroidal anti-inflammatory agent (NSAID, Figure 1) [19]. Quinazoline derivatives are employed as anticancer agents, with compounds such as gefitinib, lapatinib, dacomitinib, and various analogs being evaluated in preclinical and clinical trials [12,20,21]. In this review, we present the most significant and recent research on anti-Alzheimer’s agents featuring a quinazoline nucleus, which may aid in the design and development of novel anti-AD drugs.

## 2. Anti-Alzheimer’s Disease Agents

Alzheimer’s disease (AD) is a progressive neurodegenerative condition that causes dementia, and it is estimated that the number of patients affected by this type of dementia is constantly increasing. The prevalence of AD is estimated to be approximately 57 million individuals worldwide, due to increased life expectancy over the coming decades, with more than 150 million cases expected by 2050. AD is the most prevalent type of dementia, accounting for 60–80% of all cases [22].

**Figure 2:**
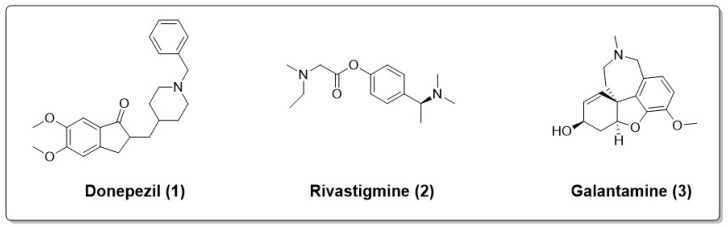
Drugs currently used in Alzheimer’s treatment that act as AChEIs.

Currently, there is no therapy for AD, a progressive neurological disorder that influences memory, reasoning, and behavior. The available treatments primarily focus on alleviating symptoms rather than decreasing the progression of the disease. These treatments include acetylcholinesterase inhibitors (AChEIs), like donepezil (**1**, Aricept), Rivastigmine (**2**, Exelon), and Galantamine (**3**, Razadyne, formerly known as Reminyl), as shown in Figure 2. These drugs work by increasing the levels of acetylcholine, a neurotransmitter essential for learning and memory, which can help mitigate cognitive symptoms and improve daily functioning to some extent. However, these medications do not address the underlying causes of Alzheimer’s, and their effectiveness can vary from person to person. Research is ongoing to find more effective treatments and ultimately a cure, but as of now, the management of Alzheimer’s remains largely symptomatic [23].

Researchers are exploring quinazoline-based compounds for their potential to inhibit key enzymes and pathways involved in the pathogenesis of AD, such as acetylcholinesterase and beta-secretase, which play critical roles in the formation of amyloid plaques and tau tangles. Additionally, quinazoline derivatives have demonstrated antioxidant activities, further supporting their potential in mitigating neuronal damage and slowing disease progression [24]. Given the complex nature of AD, research has focused on multi-target-directed ligands (MTDLs) that simultaneously address multiple disease targets, providing a more efficient approach. As a result, advancing the development of novel and enhanced therapeutic agents for treating this disorder is vital. Advancing AD research is crucial for identifying new compounds for treatment and prevention.

### 2.1. Inhibition of Cholinesterase (ChE) Enzymes 

Among the earliest hypotheses regarding the pathogenesis of AD is cholinergic dysfunction, which is characterized by a deficiency in cholinergic neurotransmission in the brain. This results from the death of cholinergic neurons and the subsequent gradual decline in the neurotransmitter acetylcholine (ACh). One of the top efficient strategies for improving the cognitive and behavioral symptoms of AD is to counteract the decline in ACh levels [23,24,25,26].

**Figure 3:**
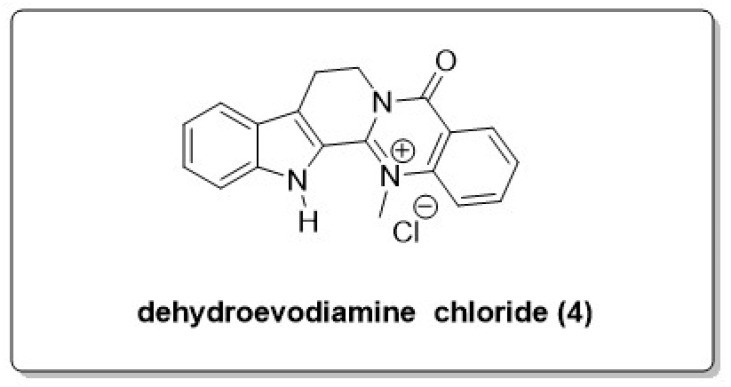
Structure of DHED hydrochloride (**4**).

In 1996, researchers discovered that dehydroevodiamine (**4**) (DHED, Figure 3), a white crystalline powder of quinazoline alkaloid extracted from Evodiae Fructus (EF), may well inhibit acetylcholinesterase (AChE) in vitro and possessed anti-dementia effects in vivo. DHED inhibited AChE in a dose-dependent manner, revealing that it is an active anti-cholinesterase constituent in EF and shows prospective as a drug candidate in the treatment of AD [27,28]. DHED is generally not available commercially. Schramm et al. developed a simple, robust, and scalable method for purifying DHED on a gram scale. Concurrently, they developed a method for the selective removal of DHE from EF extracts to facilitate the drug development and clinical use of DHED [29]. This molecule significantly reversed the scopolamine-induced dementia model, a simplified system that correlates with AD and the extent of cholinergic neuron damage. The cognitive improvement observed with DHED is attributed to increased levels of acetylcholine (ACh) in the synapse, resulting from the inhibition of AChE [30]. DHED showed a more potent anti-amnesic effect than tacrine, although its potency in AChE inhibition was lower than that of tacrine. This is attributed to the fact that DHED also possesses the ability to enhance cerebral blood flow. Fu et al. reported that DHED has a protective influence on the central nervous system and demonstrates good blood–brain barrier permeability with good antioxidant activity. Many studies assert that DHED has substantial pharmacological effects as an anti-AChE compound and improves cognitive function in rat models with memory deficits, thereby having the potential for the treatment of AD. DHED reveals robust anti-amnestic activity in vivo and reasonable AChE inhibition in vitro [31].

Other quinazoline alkaloids with reported high activity are vasicine and deoxyvasicine, extracted from the plants *Adhatoda vasica* and *Peganum harmala*, respectively. Bhanukiran et al. prepared semi-synthetic derivatives of vasicine (3-OH pyrrolidine analogs), and ex vivo studies demonstrated increased ACh levels by inhibiting AChE activity in the rat brain, thereby ameliorating memory and cognition impairment. Deoxyvasicine seems to exhibit activity against multiple targets and has shown an improvement in cholinergic neurotransmission by inhibiting AChE and activating choline acetyltransferase (ChAT) [32,33].

**Figure 4:**
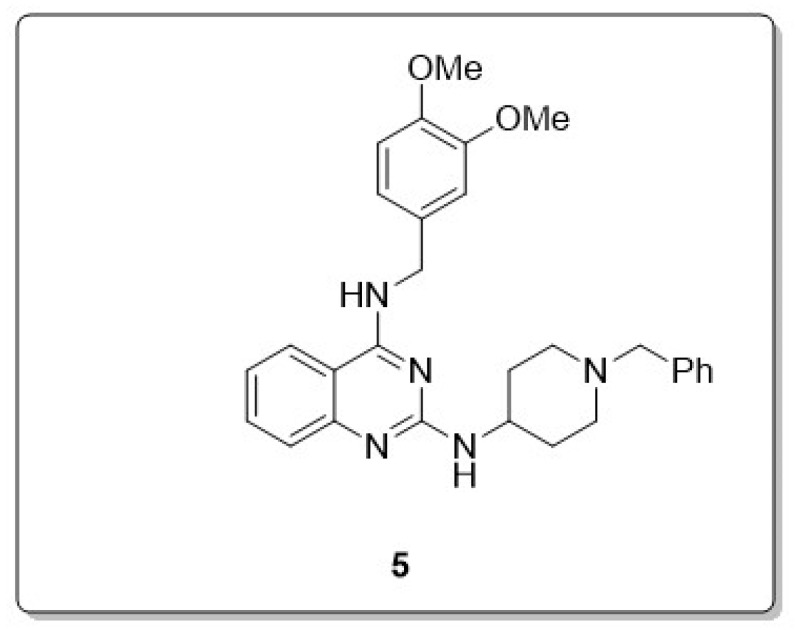
A quinazoline compound (**1**) that possesses dual activity as a cholinesterase inhibitor and Aβ inhibitor (**5**).

In the attempt to create a new ring scaffold for multi-target directed ligands (MTDLs), Mohamed et al. reported the design and synthesis of a library of 2,4-disubstituetd-quinazoline and a series of these compounds were synthesized, particularly *N*^2^-(1-benzylpiperidin-4-yl)-*N*^4^-(3,4-dimethoxybenzyl)quinazoline-2,4-diamine (**5**) (Figure 4), which exhibits dual activity in both cholinesterases (AChE and BuChE) and amyloid-beta (Aβ) inhibition (Aβ40 IC_50_ = 2.3 μM; hAChE IC_50_ = 2.1 μM; hBuChE IC_50_ = 8.3 μM). The results reported by the researchers in the study led to the discovery of quinazoline derivatives as a new class of MTDLs. Given the efficacy and potentiality of 2,4-disubstituted quinazoline derivatives for treating AD, additional research on these compounds is essential [34,35].

### 2.2. Inhibition of β-Amyloid Aggregation

Another important characteristic feature of AD is amyloid-beta (Aβ) aggregation, which is the second most accepted hypothesis for the disease’s pathology. The amyloid precursor protein (APP) is cleaved intracellularly by the proteolytic enzymes beta-secretase (β-secretase) and gamma-secretase (γ-secretase), causing the formation of the amyloid beta peptides composed of 40–42 amino acids (Aβ40/β42). The Aβ42 peptide, which has 42 amino acids, is less common but more aggregation-prone and toxic. The accumulation of Aβ, produced from hydrolysis via the amyloidogenic route, triggers neurotoxicity. The formation of plaques and tangles in the brain is associated with neuronal damage and death. The primary pathological properties of AD include brain atrophy due to regional neuronal and synaptic damage and extracellular Aβ accumulation as neuritic plaques [36,37]. In addition, Aβ accumulates in the cerebral blood vessels. Cerebral amyloid angiopathy varies in gravity, ranging from modest amounts of Aβ to significant accumulations that deform the arterial architecture and produce cortical microinfarcts. Aβ deposition is thought to begin 20 years before the onset of clinical symptoms. Based on the amyloid hypothesis, the deposition of Aβ plaques in the brain leads to synaptic dysfunction and neurodegeneration. The primary factor promoting AD is the malfunction in the processes that regulate the formation, accumulation, or elimination of Aβ [38,39].

**Figure 5:**
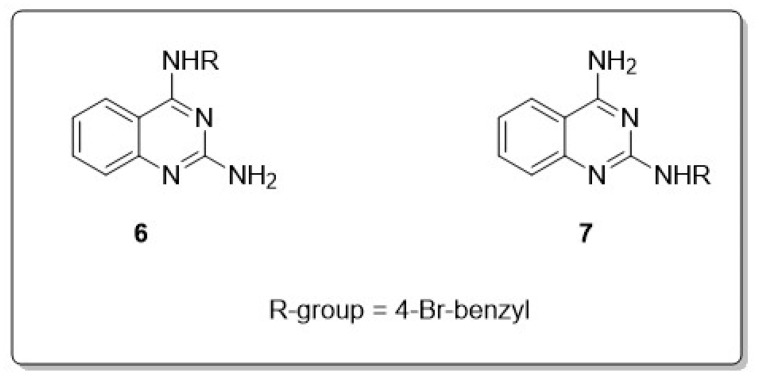
Inhibition data for DAQ isomeric derivatives (**6** vs. **7**).

Toward the discovery of novel Aβ aggregation inhibitors as anti-AD agents, researchers are exploring various compounds and mechanisms to prevent the formation of Aβ plaques and alleviate neurotoxicity, including both peptidic and non-peptidic molecules. In this field, quinazoline compounds have shown promise. Mohamed et al. synthesized 34 analogs of 2,4-diaminoquinazolines (DAQs) and explored their activity against the Aβ40/Aβ42 accumulation kinetic. It was shown in this research that the anti-Aβ activity is affected by the isomeric location of substituents at the 2- or 4-position of the quinazoline amine template; along with that, these compounds constitute a new class useful for designing small molecules with anti-amyloid aggregation properties. They discovered nine DAQs that showed anti-Aβ aggregation properties either exceeding or matching those of the reference agents curcumin, orange G, and resveratrol. Halogen-substituted benzyl groups usually revealed a superior anti-Aβ accumulation activity, with the *N*^4^-isomer furnishing better selectivity for Aβ40; on the other hand, the *N*^2^-isomer demonstrated better inhibition of Aβ42 accumulation. The *N*^4^-isomer (**6**) with a 4-bromobenzyl substituent was recognized as the most potent Aβ40 aggregation inhibitor (IC_50_ = 80 nM), while the related *N*^2^-isomer (**7**) was the most potent Aβ42 accumulation inhibitor (IC_50_ = 1.7 µM), as shown in Figure 5 [40].

**Figure 6:**
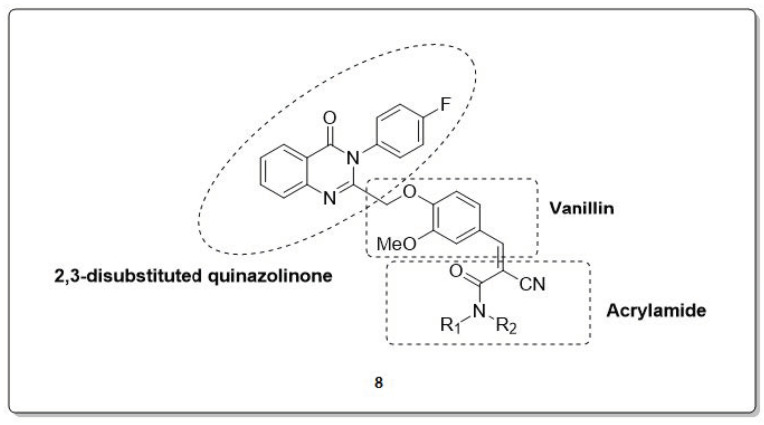
2,3-disubstituted quinazolinone and vanillin acrylamide hybrids (**8**).

Rao et al. recently reported that 2,3-disubstituted quinazoline derivatives with vanillin acrylamide act as novel MTDLs (**8**, Figure 6). These compounds modulate amyloidogenic assembly, inhibit cholinesterase enzymes, exhibit antioxidant activity, and provide neuroprotective effects. The synthesized compounds were found to possess potent activities such as MTDLs [41].

### 2.3. Targeting Tau Protein

Under normal conditions, tau protein is primarily found in neurons, where it stabilizes microtubules. In Alzheimer’s disease, tau protein becomes abnormally modified, leading to several pathological processes, such as excessive phosphorylation. Hyperphosphorylation decreases tau’s affinity for microtubules, causing it to detach and destabilize them. Detached tau proteins start to aggregate, forming insoluble fibrils. These fibrils then coalesce into neurofibrillary tangles (NFTs) within neurons. NFTs disrupt the normal functioning of neurons. The formation of NFTs and the loss of microtubule stability result in impaired intracellular transport, which is critical for neuronal function and survival. This leads to synaptic dysfunction, neuronal death, and the progressive loss of cognitive functions associated with AD. Targeting tau protein in AD treatment focuses on several strategies due to the protein’s involvement in neurofibrillary tangles, a hallmark of the disease. These strategies include tau aggregation inhibitors, tau clearance enhancement, microtubule stabilization, and the inhibition of tau protein phosphorylation [42,43,44].

**Figure 7:**
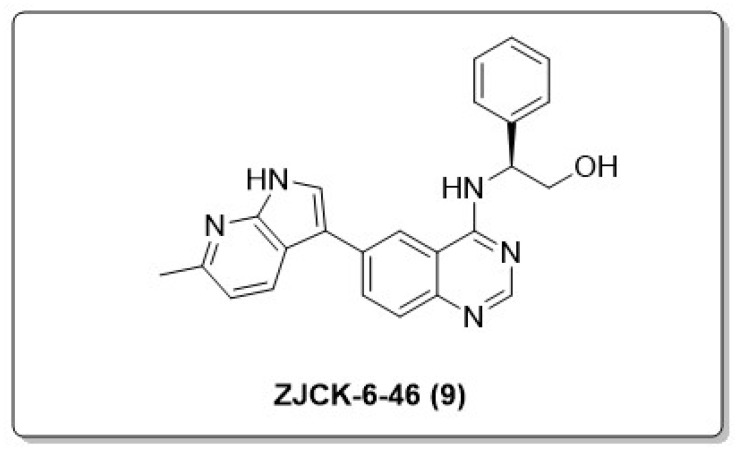
Novel DYRK1A inhibitor ZJCK-6-46 (**9**).

Via rational design, structural optimization, and holistic evaluation, Chen et al. identified **ZJCK-6-46** (**9**) as the most potent DYRK1A inhibitor, with an IC_50_ value of 0.68 nM. **ZJCK-6-46** exhibited satisfactory in vitro ADME (absorption, distribution, metabolism, and excretion) properties. Additionally, **9** exhibited favorable bioavailability and blood–brain barrier permeability. In vivo investigations confirmed that **9** has the potential to ameliorate cognitive dysfunction by significantly reducing phosphorylated tau levels and neuronal cell death. Exhibiting low acute toxicity, the compound also presents minimal risk of acute liver and kidney injury. Researchers suggest that **9** could serve as a valuable molecular tool for exploring treatment strategies and understanding the mechanisms underlying AD and its pathogenesis, facilitating the development of anti-AD drugs (Figure 7) [45].

**Figure 8:**
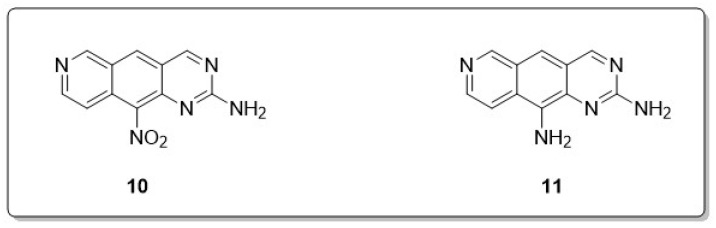
The quinazoline derivatives (**10** and **11**) displayed nanomolar-level potency toward CLK1 and/or DYRK1A.

According to Esvan et al., a series of pyrido [3,4-g]quinazolines, a type of aza-heterocyclic compound, were synthesized and evaluated for their inhibitory effects on CDK5, CK1, GSK3, CLK1, and DYRK1A. Among the compounds tested, two derivatives (**10** and **11**) exhibited significant potential as inhibitors of the CLK1 (**10**) and DYRK1A (**11**) kinases, as shown in Figure 8 [46].

### 2.4. Antioxidant Activity

Oxidative stress is a critical factor in the pathogenesis and progression of AD. It refers to an imbalance relating to the formation of reactive oxygen species (ROS) and the brain’s capacity to scavenge these reactive intermediates or repair the resulting impairment. In AD, excessive ROS may be able to harm cellular components, involving lipids, proteins, and nucleic acids, contributing to neuronal dysfunction and loss. Research involving ex vivo and in vivo models has shown that antioxidant treatments can provide substantial protective benefits [47,48]. Notably, compounds containing several phenol units (polyphenols) like curcumin, resveratrol, and quercetin have demonstrated prospective efficacy for the treatment of AD [49,50].

**Figure 9:**
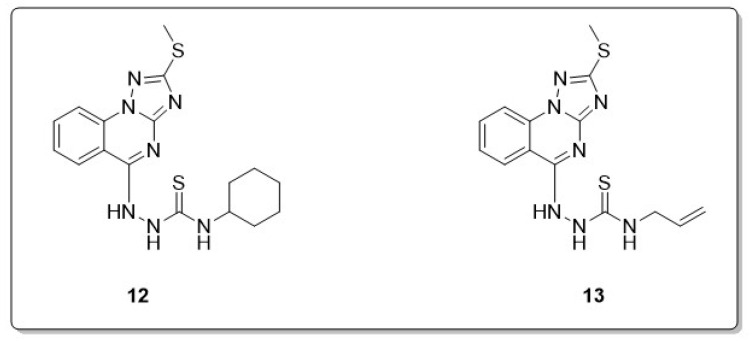
Antioxidant-active triazoloquinazolines (**12**, **13**).

Ongoing research and interdisciplinary collaboration are essential for discovering effective remedies to counteract the harmful effects of free radicals. Another research group designed and synthesized triazoloquinazoline derivatives. Density functional theory was analyzed using the bond dissociation energy values of the active NH groups in various compounds. The findings indicated that a higher number of transferred hydrogen atoms improves the free radical scavenging endeavor of these compounds. As a result, compounds **12** and **13** exhibited the maximum activity in this series (Figure 9). The antioxidant activity observed in the investigated compounds (**12** and **13**) may be associated with the presence of hydrazine and isothiocyanate moieties [51].

### 2.5. Multi-Target-Directed Ligands (MTDLs)

Multi-target-directed ligands (MTDLs) are important in developing novel anti-Alzheimer’s drugs because they can simultaneously address multiple pathological processes associated with the disease, such as Aβ plaques, tau tangles, oxidative stress, and cholinesterase enzymes. This approach may help reduce the continuous failure in finding new drugs. By targeting multiple disease mechanisms simultaneously, MTDLs can potentially overcome the limitations of single-target therapies, which often fail to address the complex and multifaceted nature of diseases like AD. This comprehensive approach not only enhances therapeutic efficacy by targeting various aspects of the disease but also reduces the risk of drug resistance and potential side effects associated with polypharmacy. By integrating multiple mechanisms of action into a single compound, MTDLs offer a promising strategy for more effective and holistic treatment of AD [52,53].

**Figure 10:**
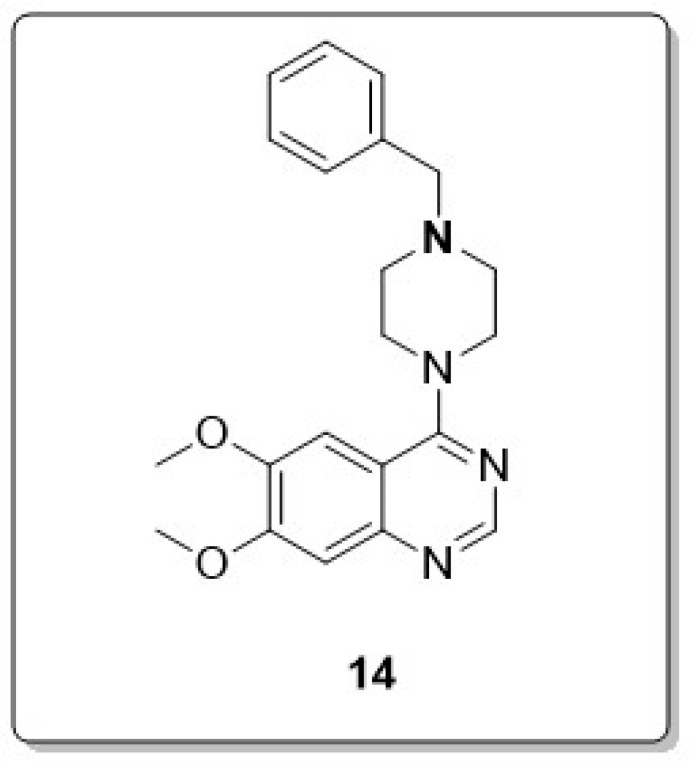
Structure of quinazoline derivative as MTDLs (**14**).

Verma and coworkers, considering the multifaceted nature of the disease, rationally designed and synthesized a series of new quinazoline derivatives, introducing different substituted piperazines at the carbon-4 position of quinazoline bearing in mind a bioisosteric replacement for N-benzylpiperidine ring, a pharmacophore structure characteristic of the Food and Drug Administration (FDA)-approved drug donepezil. The inhibitory activities of these derivatives were biologically tested against hChE and Aβ aggregation. The biological evaluation against hChE and hBACE-1 showed that the quinazoline derivative **14** possesses the most potent activity (hAChE, IC50: 0.283 μM; hBChE, IC50 > 10 μM; hBACE-1, IC_50_: 0.231 μM). And, **14** also exhibited Aβ accumulation inhibition potential with non-neurotoxic liabilities and BBB permeability, as shown in Figure 10 [54].

In the past five years, research interest in quinazolines has grown significantly due to their diverse biological activities, including acetylcholinesterase inhibition, antioxidant properties, and modulation of amyloid-beta aggregation. Advances in synthetic methodologies and the growing focus on multi-target drug design have further highlighted the potential of quinazolines as innovative neurodegenerative disease therapies. While significant progress has been made, further research is necessary to fully explore their therapeutic potential and optimize their clinical applications [55,56].

## 3. Conclusions

The quinazoline nucleus has emerged as a highly promising scaffold for the design and synthesis of anti-AD drugs due to its multifaceted pharmaceutical activities. The versatility of the quinazoline scaffold enables the incorporation of diverse functional groups, enhancing its potential to modulate key processes involved in the pathogenesis of AD, such as cholinergic deficit, β-amyloid aggregation, tau protein hyperphosphorylation, and oxidative stress. Studies have demonstrated that quinazoline derivatives exhibit significant inhibitory activity against cholinesterase enzymes implicated in the breakdown of acetylcholine, thereby ameliorating cholinergic transmission deficits observed in AD patients. Furthermore, quinazoline-based compounds have shown potential in inhibiting the Aβ aggregation pathway, thus reducing the formation of neurotoxic β-amyloid plaques. In addition, quinazoline derivatives have been found to possess antioxidant properties, mitigating oxidative stress and reducing neuroinflammation, both of which are key contributors to neuronal damage in AD. These multifaceted actions underscore the potential of quinazoline scaffolds to target multiple aspects of AD pathology, offering a promising avenue for the development of MTDLs.

## Data Availability

Not applicable.
